# New Insights into Regulatory T Cells: Exosome- and Non-Coding RNA-Mediated Regulation of Homeostasis and Resident Treg Cells

**DOI:** 10.3389/fimmu.2016.00574

**Published:** 2016-12-06

**Authors:** Peiyao Li, Changhong Liu, Zhibin Yu, Minghua Wu

**Affiliations:** ^1^The Key Laboratory of Carcinogenesis of the Chinese Ministry of Health, Xiangya Hospital, Central South University, Changsha, Hunan, China; ^2^The Key Laboratory of Carcinogenesis and Cancer Invasion of the Chinese Ministry of Education, Cancer Research Institute, Central South University, Changsha, Hunan, China; ^3^Hunan Key Laboratory of Non-resolving Inflammation and Cancer, Disease Genome Research Center, The Third Xiangya Hospital, Central South University, Changsha, Hunan, China

**Keywords:** Treg cell, exosome, non-coding RNA, resident Treg cell, Treg homeostasis

## Abstract

Regulatory T (Treg) cells are a group of cells that are heterogeneous in origin and in functional activity. Treg cells comprise a necessary balance to adaptive immune responses. As key regulators of self-tolerance, Treg cells have been involved in a series of pathologic processes and considered as therapeutic targets. Here, we summarize recent research regarding Treg cell origins and their functional classification, highlight the role of exosomes and non-coding RNA in modulating Treg cell homeostasis, and discuss the current understanding of resident Treg cells.

## Introduction

Immune tolerance regulation is a critical aspect of immunology. Distinct populations of T cells with suppressor functions make a major contribution to such regulation. Regulatory T (Treg) cells are important for preventing inappropriate responses by the immune system (1). Treg cells exert their suppressive role from triggering of innate immune cells to adaptive cell-mediated responses. Treg cells have been implicated in a number of pathologic processes involving severe systemic autoimmunity and many malignancies (2, 3). As critical regulator of immune tolerance and homeostasis, Treg cells have been regarded as immunotherapeutic targets. Manipulation of the number and/or suppressive activity of Treg cells has been shown to be impactful in the treatment of autoimmune disorders, allograft rejection, and cancer (4–6). Thus, understanding the local immune regulation and regulatory mechanisms of Treg cells is essential. In this review, the characteristics of Treg cells and tissue-resident Treg cells are summarized. The regulatory mechanisms of Treg cells are also discussed, focusing on exosomes and non-coding RNA.

## Characterization of Treg Cells

Regulatory T cells are distinguished from other lymphocytes by several characteristics, including surface marker, transcription factor, origin, and function (Table 1). Treg cells express CD25 (IL-2 receptor α chain) and are dependent on stimulation by IL-2 for their function (7). Foxp3 was discovered to be a “master regulator” of Treg cell development and function (8–11). Mutations of the Foxp3 gene in humans result in Treg deficiencies and are responsible for immunodysregulation polyendocrinopathy enteropathy X-linked syndrome (12). Foxp3 and CD25 are reliable and constitutive markers that have been used to isolate and characterize Treg cells. In addition to CD25 and Foxp3, Treg cells express co-stimulatory and co-inhibitory molecules that are involved in their suppressive function, such as CD28 and cytotoxic T lymphocyte antigen 4 (CTLA4), tumor necrosis factor (TNF), and TNF receptor family members, including RANKL and GITR, and Toll-like receptors (4). It has been shown that CD127 expression inversely correlates with Foxp3 expression and CD4^+^ Treg cells suppressive function (13) and that the combined use of Foxp3^+^, CD25^+^, and CD127^−^, might better define the Treg cell population with suppressive functions (14).

**Table 1:** **Markers for Treg cell subsets**.

Treg cell subsets				
Origin subsets		Functional subsets		
Thymus-derived Treg	Peripherally derived Treg	Resting Treg	Effector Treg	Tissue-resident Treg
Cytotoxic T lymphocyte antigen (CTLA4)	CTLA4	CTLA4^low^	CTLA4^hi^	Adipose tissue-resident Treg
GITR	GITR	CD62L^hi^	CD62L^low^	PPARGγ^hi^, Foxp3^hi^
CD103	CD103	CD45RA^hi^	CD45RA^low^	
Helios	CD25^hi^	CD25^hi^	CD44^hi^	
Neuropilin-1	CD127^low^	CD127^low^	KLRG1^+^ CD103^+^	Skeletal muscle-resident Treg
TIGIT	Foxp3^hi^	CCR7^hi^	CD25^hi^	Tbet^hi^, Foxp3^hi^, CXCR3^hi^
FCRL3			CD127^low^	
CD25^hi^			Foxp3^hi^	
CD127^low^			CCR7^low^	
Foxp3^hi^			CTLA4^hi^	

Treg cells can be separated according to their two possible origins: tTreg (thymus-derived Treg) cells and pTreg (peripherally derived Treg) cells, also called natural Treg cells and induced Treg cells, respectively (15). Most Treg cells arise in the thymus, where the expression of Foxp3 is initiated *via* a combination of self-antigen recognition with moderate- to high-avidity and microenvironmental influences, and these tTreg cells migrate to the periphery to maintain self-tolerance (16). Moreover, tTreg cells can also be induced in the periphery from Foxp3^–^ recent thymic emigrants (17). Another way of Treg generation is in the periphery, where CD4^+^ T cells develop into pTreg cells upon encountering antigens under certain conditions (18, 19). Two populations of peripherally induced CD4^+^ Treg cells have been described: Tr1 cells and Th3 cells, they are induced in peripheral, secrete interleukin 10 (IL-10) and/or transforming growth factor beta (TGF-beta), and exert suppress function *via* a cytokine-dependent mechanism (20–22). Both thymic-derived and peripherally induced Treg cells are antigen specific, possess T-cell receptors, and are selected with a suppressive function. A variety of molecular markers can be used to distinguish different Treg populations. Transcription factor Helios and cell surface glycoprotein neuropilin-1 are usually highly expressed by tTreg cells but poorly expressed by pTreg cells, as thus, both these molecular markers can be applied to distinguish tTreg from pTreg cells; nevertheless, pTreg cells may upregulate these factors expression depending on local inflammatory conditions or the type of antigen-presenting cells and activation signals that are present (15, 23, 24). Furthermore, a study of human Treg subsets described an important role for T cell immunoreceptor with Ig and ITIM domains (TIGIT) and FcR-like 3 (FCRL3) in distinguishing tTreg cells from pTreg cells (25).

Regulatory T cells can also be divided into functional subpopulations as well as into origin subsets (26–28). (1) Resting Treg cells (CD62L^hi^CCR7^+^ or CD45RA^hi^CD25^low^ Treg cells), also known as central or naive Treg cells, conprise the great number of Treg cells in secondary lymphoid organs and in circulation. Resting Treg cells have a history of antigen exposure and baseline suppressive function, and they share circulation patterns and activation markers with naive and memory conventional T cells. (2) Effector Treg cells (CD45RA^low^CD25^hi^ or CD62L^low^CCR7^low^CD44^hi^KLRG1^+^CD103^+^ Treg cells), also known as activated Treg cells, constitute a small part of Treg cells in circulation and in secondary lymphoid organs (29). This subset has enhanced function and signs of recent antigen encounter and shares phenotypic features with activated conventional T cells. It remains unclear whether effector Treg cells are capable of reverting to resting Treg cells or are terminally differentiated. (3) Recently, a greater emphasis has been placed on a specific subset of tissue-resident Treg cells that take part in immune processes as well as in the maintenance of tissue homeostasis (27, 28, 30, 31). The phenotype and function of tissue-resident Treg cells are different from those of the classical lymphoid Treg cells. Each tissue might have its own unique tissue-resident Treg cells, which have good sensitivity and a high turnover rate in response to a number of environment signals (30). These characteristics of tissue-resident Treg cells enable rapid adjustments in Treg cell location and number that are required to effectively react to immune dynamics (27, 30). Moreover, to be able to optimally control the immune response in dynamic tissue microenvironments, Treg cells can afford a certain degree of functional plasticity. Treg cells preserve their core immunosuppressive characteristics and alter their transcriptional program to achieve functional plasticity. Recent work has demonstrated that tissue-resident Treg cells often have distinct transcription programs from lymphoid organ Treg cells. For instance, visceral adipose tissue Treg cells show high expression of the transcription factor peroxisome proliferator-activated receptor γ, which acts as a crucial regulator of adipocyte differentiation. Similarly, skeletal muscle-resident Treg cells display a transcriptional program that sustains their repair function following acute injury (32). Furthermore, to control the Teff cell response, Treg cells can express distinct transcription factors and immunosuppressive molecules associated with that type of Teff cell. For example, Tbet^+^ Treg cells, induced by type 1 inflammatory conditions, express chemokine (C–X–C motif) receptor 3 and accumulate at T helper 1 (Th1) cell-mediated inflammation sites. CXCR3 is a key molecule on Th1 cells that mediates the accumulation of Th1 cells at sites of local inflammation. Thus, the function of Treg cells partially depends on the degree of plasticity that they exhibit in response to the microenvironment (32–34).

## Treg Cells and Exosomes

Exosomes are small membrane vesicles derived from multivesicular bodies or from the plasma membrane (35). Exosomes play critical roles in intercellular communication, as they transfer RNAs, proteins, and other type of molecules between donor and receptor cells (36). Exosome protein contents mostly reflect that of the parent cells, and exosomes are enriched in cytoskeleton molecules, cytoplasmic enzymes, signal transduction proteins, and so on (37). Furthermore, exosomes contain a variety of non-coding RNAs (ncRNAs), involving microRNAs (miRNAs), long non-coding RNAs (lncRNAs), and circRNAs (36–39).

**Figure 1:**
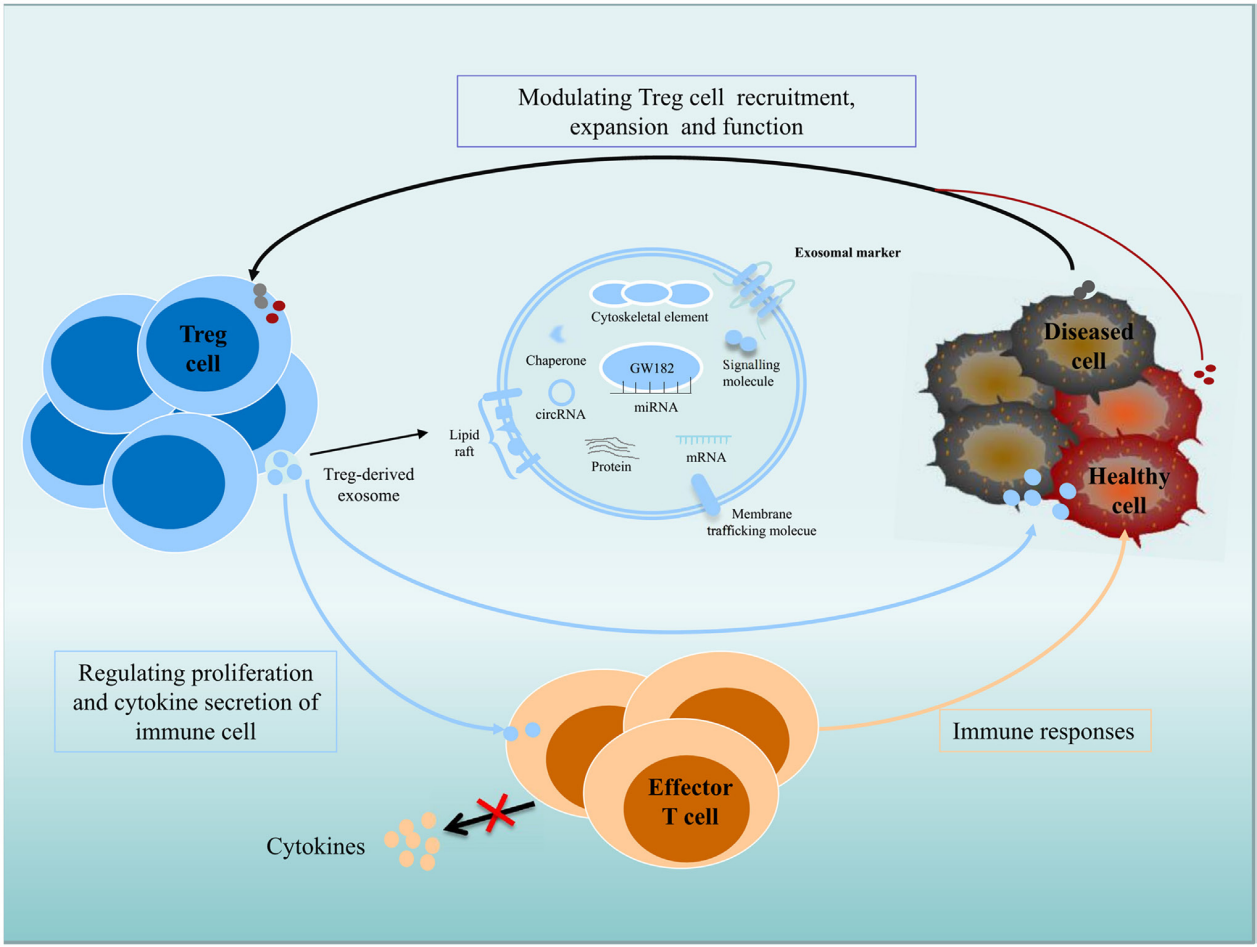
**Role of exosomes in intercellular communication between Treg cells and recipient or donor cells**. Exosomes transfer their contents, including proteins, lipids, and RNAs, between cells. Immune and non-immune cell-derived exosomes have important roles in the regulation of immunity. Exosomes contribute significantly to the function of Treg cells, and Treg cell-derived exosomes can be delivered to immune cells and diseased or healthy tissue cells; regulate the proliferation and cytokine secretion of effector T cells; and modulate the immune response. Diseased cells, including tumor cells, can modulate Treg cell recruitment, expansion, and function *via* an exosome-based pathway.

Exosomes participate in important biological functions and are involved in numerous physiological processes. Immune and non-immune cell-derived exosomes play critical roles in immunity regulation; these exosomes can mediate immune homeostasis and can drive inflammation, autoimmunity, and infectious disease pathology (40–44). Exosomes derived from Treg cells appear to be greater in quantity than those from other type of T cells and are regulated by changes in intracellular calcium, synthesis of the sphingolipid ceramide, hypoxia, and the presence of IL-2 (45–48). Exosomes make great contribution to Treg cells function (Figure 1), as inhibiting the release of exosomes can reverse the suppressive capabilities of Treg cells, such as effector T cells (Teffs) suppression and disease prevention. Rab27-DKO Treg cells that failed to release exosomes also failed to prevent disease, resulting in colon shortening, weight loss, and increased IFNγ expression. The failure of these cells to control Teffs also led to significant colonic and systemic inflammation and IFNγ expression (49). Recently, the transfer of miRNAs, including Let-7d, Let-7b, and miR-155, *via* Treg cell exosomes to conventional T cells has been shown. Let-7d-containing Treg cell-derived exosomes contributed to the suppression of Th1 cell proliferation and IFNγ secretion *via* Cox-2 (40). However, upregulation of Let-7b in Th1 cells had little impact on proliferation or IFNγ production. Treg cells transfer miR-155 to conventional T cells with a concomitant upregulation of several Treg-cell-associated genes in recipient cells (40). Furthermore, several immunomodulatory molecules, including CD73, CD25, and CTLA4, were also found in Treg exosomes (43, 49). Treg cell-derived CD73-expressing exosomes contribute to their suppressive activity through the production of the anti-inflammatory mediator adenosine (43). Adenosine can bind to adenosine receptors, triggering intracellular cAMP, leading to the inhibition of cytokine production by activated Teffs. CD25 and CTLA4 are expressed in Treg cell-derived exosomes, but these molecules may not contribute to the suppressive function of Treg cells. Several molecules, including FasL, CD39, PDL1, and Galectin1, are present on exosomes derived from other type of cells. FasL- and CD39-containing exosomes have been shown to have immunomodulatory properties. Lymphoblastoid cell-derived MHCII^+^FasL^+^exosomes induced apoptosis in CD4^+^ T cells. Tumor exosomes express CD39 and CD73, which are capable to suppress T cells (50–53). Whether they are also present on Treg cell-derived exosomes and play a role in Treg cells suppressive function is yet to be validated.

In addition, recent studies have investigated that exosomes derived from tumor cells exert widespread detrimental effects on the immune system (54). Secreted exosomes can serve as signaling tools in mediating tumor cell–Treg cell communication (55). In nasopharyngeal carcinoma, tumor cell-derived exosomes facilitated the expansion of Treg cells and upregulated their suppressive functions. The tumor cell-derived exosomes also promoted the conversion of conventional CD4^+^CD25^−^ T cells into Treg cells and enhanced the chemoattraction of Treg cells through CCL20 (54). Extracellular vesicles derived from colorectal cancer cell induced a phenotypic change of T cells to Treg-like cells, which had remarkable tumor-growth promoting activity by activating TGF-β/Smad signaling and inactivating SAPK signaling (56). In lung carcinoma, tumor-derived miR-214 reduced PTEN expression (phosphatase and tensin homolog) and promoted the expansion of Treg cells, and miR-214-induced higher secretion of IL-10 in Treg cells and promoted tumor growth (55). It is possible that cancer cells can actively control the immune cells antitumor activities by transfer tumor-specific molecules to recipient immune cells, including Treg cells, *via* an exosome-based pathway (55). Clearly, exosomes are vital mediators of immunity, for which there will be extensive therapeutic applications (36, 49).

## Treg Cells and Non-Coding RNAs

**Figure 2:**
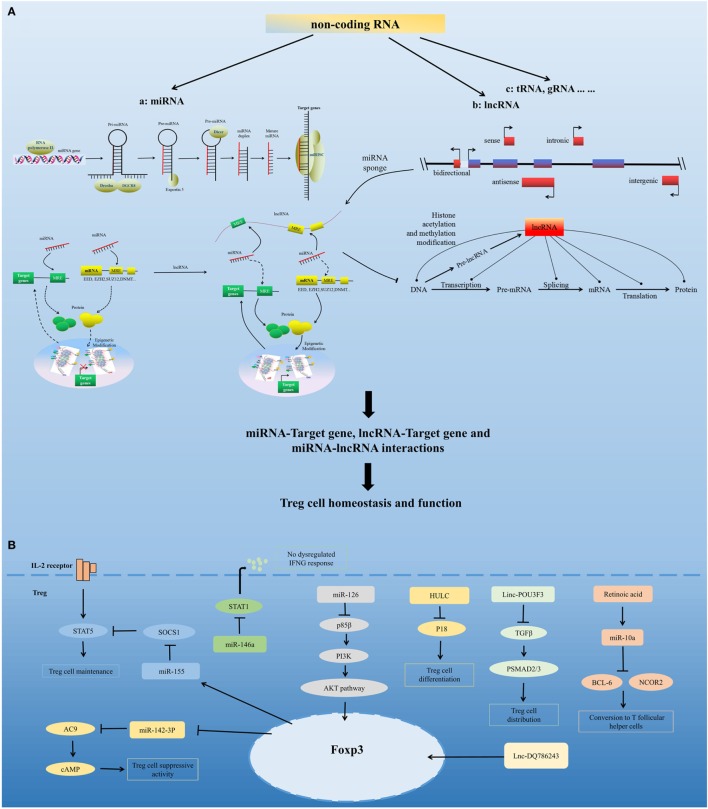
**Non-coding RNA-mediated regulation of Treg cell homeostasis and function**. **(A)** Model for non-coding RNAs (ncRNA)-mediated regulation of Treg cell homeostasis and function. ncRNAs include highly abundant and functionally important RNAs, such as microRNAs (miRNA), long non-coding RNAs (lncRNA), and tRNA. miRNAs are sequentially processed from longer transcripts by the RNase III enzymes Drosha and Dicer. Pri-miRNAs are processed by Drosha into hairpin structures (pre-miRNAs). Exportin 5 shuttles pre-miRNAs from the nucleus into the cytoplasm, where the RNase III Dicer cleaves off the hairpin loop of the pre-miRNA. The duplex segregates, and the mature single-stranded miRNA associates with argonaute proteins and other accessory proteins to form the miRNA-induced silencing complex, which directly mediates the translational repression and the increased degradation of its mRNA targets. miRNA can also indirectly regulate gene expression by repressing the expression of several key enzymes involved in epigenetic modification processes, such as DNA methylation and histone modification. Based on the position of lncRNA relative to the neighboring protein-coding genes in the genome, lncRNAs can be divided into five categories, namely, sense, antisense, bidirectional, intronic, and intergenic. lncRNAs can modulate chromatin modification, mRNA stability, miRNA activity, and the function of proteins by interacting with chromatin, RNA, and protein. lncRNA functions as a miRNA sponge, sequestering miRNAs to regulate the expression level of other transcripts sharing common miRNA response elements. This process leads to fewer miRNA molecules available to bind to target mRNA and, thus, an increase in its protein expression level. miRNA, lncRNA, and mRNA form a well-regulated interacting network and play critical regulatory roles in Treg cell homeostasis and function. **(B)** Regulation of Treg cells by several representative miRNAs and lncRNAs, including miR-155, miR-146a, miR-126, miR-10a, miR-142-3p, HULC, Linc-POU3F3, and Lnc-DQ786243.

Non-coding RNAs comprise multiple classes of RNA transcripts that are not transcribed into proteins but have been shown to regulate the transcription, stability, or translation of protein-coding genes. To date, there have been many studies of miRNAs and lncRNAs, and other classes of experimentally identified ncRNAs with various lengths and characteristics have also been reported (57). ncRNAs are key regulators of the immune system and regulate important aspects of Treg cells, including Treg cells development, homeostasis, and function. Dynamic homeostatic processes maintain the diverse pool of Treg cells and preserve their number in a normal range. Treg cells can tailor their functions and homeostatic properties to a wide range of conditions (27). Treg homeostasis and function is governed by a number of factors; here, we discuss miRNA- and lncRNA-mediated regulation of Treg cell homeostasis and function (Figure 2).

## miRNA-Mediated Regulation of Treg Cell Homeostasis and Function

MicroRNAs are a group of evolutionarily conserved small non-coding RNAs. They carry out their function by guiding the miRNA-induced silencing complex to target mRNAs (58, 59). miRNAs can directly regulate the expression of target genes by sequence-specific binding to the 3′ untranslated region (3′ UTR) or other regions, and they can also indirectly regulate gene expression by repressing the expression of several key enzymes involved in epigenetic processes, such as DNA methylation and histone modification (60, 61). Research has confirmed the requirements for miRNA expression in Treg cells and shown that miRNAs are important for the maintenance of Treg cell homeostasis and their immunosuppressive function (62).

Studies have shown that depletion of thymus-miRNAs downregulated the number of Treg cells in the thymus, lymph nodes, and spleen, with normal development of conventional T cells in the thymus (63). miRNA-deficient CD4^+^ T cells fail to develop into tTreg cells and have reduced potential to differentiate into iTreg cells (62). Dicer-deficient Treg cells showed inferior proliferative potential, impaired suppressor function, and impaired peripheral homeostasis. Foxp3 downregulation interrupts Treg cell lineage stability in Dicer-deletion mice (64, 65). Similar to Dicer, deletion of Drosha in Treg cells leads to defective suppressive activity (66). In addition, Treg cell-specific miRNA-deficient mice show fatal early-onset lymphoproliferative syndrome, and the conditional deficiency of Dicer or Drosha in Foxo3^+^ Treg cells gives rise to the early onset of severe spontaneous autoimmunity (64–66). These findings confirmed the critical role of miRNA in Treg cell development and function and in preventing immune disease.

Several miRNAs have been found that affect Treg cell homeostasis and function. miR-155 is highly expressed in Treg cells. Foxp3 binds to the B cell integration cluster (encodes the primary miR-155 transcript), and controls this high expression of miR-155 in Treg cells (67–70). Recent studies have shown that miR-155 contribute to the development and homeostasis of Treg cell, but not the function of Treg cell. miR-155 deletion mice show a decreased number of tTreg cells and pTreg cells, due to defective development (63). miR-155 facilitates Treg cell homeostasis by targeting suppressor of cytokine signaling 1 (69), a negative regulator of signal transduction and activation of transcription (STAT) 5 that has a crucial role in the IL-2 signaling pathway and in Treg cell development. miR-155 deletion also downregulates the IL-2 production of CD4^+^ T cells (71), which demonstrates that miR-155 might control IL-2-directed Treg cell homeostasis *via* both cell-intrinsic and cell-extrinsic pathway (62). miR-10a contributes to Treg cell stability by maintaining high levels of Foxp3 expression. TGF-beta and retinoic acid, which boost the Treg cell phenotype, are need for maximal induction of miR-10a in pTreg cells (72, 73). In addition, miR-10a inhibits pTreg cells conversion into T follicular helper cells by directly targeting BCL-6 and its co-repressor NCOR2. The expression of miR-10a in Treg cells is inversely correlated with susceptibility to autoimmune disease (74, 75). miR-146a has a marked effect on Treg cell function and plays a critical role in Treg cell-mediated immunological tolerance. miR-146a-deficient mice developed severe lympho- and myeloproliferative syndrome (76) and had an elevated number of Treg cells in the periphery that had a modest increase in activation markers and heightened proliferative activity. The restriction of miR-146a deficiency mainly to Treg cells resulted in IFNγ-dependent immune-mediated lesions and a Th1 cell-mediated pathology (77, 78), which was similar to the disease observed in Treg cell-specific Dicer- or Drosha-deficient mice. In addition, miR-146a controls Treg-mediated suppression of IFNγ-dependent Th1 responses and inflammation by targeting STAT1 (78). miR-126 is highly expressed in Treg cells. Silencing of miR-126 could attenuate the suppressive activity of Treg cells. miR-126 regulates the induction and function of Treg cells through the p85β/PI3K/Akt pathway. miR-142-3p may be a unique molecule in Treg cell function. It has been found that Treg cells exert their suppressor function by transferring cAMP to responder T cells (79). miR-142-3p restricts cAMP production in Treg cells by targeting adenylate cyclase 9 (AC9), whereas Foxp3 could maintain the activity of the AC9/cAMP pathway by downregulating miR-142-3p in Treg cells (80). In addition to the miRNAs mentioned above, many other miRNAs play important roles in the regulation of Treg cells, including miR-21 (81), miR210 (82), miR15a/16 (83), among others.

## Regulation of Treg Cells by lncRNAs

Long non-coding RNAs are transcripts of more than 200 bp that are often expressed with higher cell specificity than protein-coding genes despite having lower expression levels. Recent studies have found that several lncRNAs can affect Treg cells. The lncRNA HULC, which is upregulated in hepatocellular carcinoma, affects the differentiation of Treg cells by downregulating the level of p18 directly in HBV-related liver cirrhosis (84). Linc-POU3F3 was able to facilitate the distribution of Treg cells among peripheral T cells, which caused increased cell proliferation of gastric cancer cells through recruiting TGF-beta and activating TGF-beta pathway (85). The lncRNA DQ786243 affects the expression of cAMP response element binding protein and Foxp3 by Treg cells in Crohn’s disease (86). Further studies are needed to identify the mechanisms of lncRNAs in the regulation of Treg cells.

## Tissue-Resident Treg Cells

Regulatory T cells are present in various non-lymphoid tissues in health and disease. Each tissue might have its own unique tissue-resident Treg cells, and the phenotype and function of tissue Treg cells are different from those of classical lymphoid Treg cells (30). These cells not only display some activated and/or effector cell features but also show some unique properties, such as specific chemokine receptors, transcription factors and adhesion molecules or distinct T cell antigen receptor repertoires, mechanisms of action, targets, and migration patterns (23). Treg cells have been found in several non-lymphoid tissues, including adipose tissue, skeletal muscle, intestinal mucosa, skin, and tumor tissue (30, 87–89). Understanding the development and the maintenance of tissue-resident Treg cells provides important insights into local immune regulation and tissue-specific biological therapies. Here, we review the current state of knowledge of intestine-resident Treg cells and tumor tissue-resident Treg cells.

## Intestine-Resident Treg Cells

There are a large number of Treg cells in the intestines because of the exposure to food-derived antigens and commensal microflora. Intestine-resident Treg cells are different from other organ Treg cells and have intestine-specific phenotypes, TCR repertoires, and functions (90, 91). High levels of microbe-derived TLRs and metabolites of the commensal flora can dramatically influence the development, function, and maintenance of intestine-resident Treg cells (19). In addition, specialized CD103^+^ DCs, together with TGF-beta and retinoic acid, can enhance pTreg cell development in the intestine and also induce Treg cells to express intestinal homing receptors (92, 93). Intestine-resident Treg cells are a self-renewing and stable population, with a few proportions derived from the periphery after their initial development and seeding early in life. Further understanding of the molecular mechanisms responsible for the tissue-specific and condition-adapted development of stable Treg cell populations in the intestines could supply new treatment approaches for many diseases (91).

## Tumor-Resident Treg Cells

Depressed cellular immunity has been demonstrated in patients with a variety of lymphoreticular and non-lymphoreticular neoplasms. Given the recent successes of immunomodulatory antitumor strategies, there is growing interest in the more heterogeneous group of tumor-infiltrating Treg cells (30, 94). Elevated percentages of Treg cells are found in the total T cell population isolated from tumor tissue, and these cells can account for 30–50% of CD4^+^ T cells, depending on the tumor type (95). Tumor-resident Treg cells have been identified and characterized, and, similar to tissue-resident Treg cells, the phenotypes of tumor-resident Treg cells are different from those found in lymphoid organs or in the circulation. Foxp3^+^CD4^+^ T cells from the tumor environment show upregulation of cell-surface markers, including CTLA4, TIM3, and PD1, as well as a variety of chemokine receptors and suppressive cytokines. Tumor-resident Treg cells represent an important cellular mechanism by which tumors evade immunosurveillance, as these cells are capable of restricting the proliferation and cytokine production of a wide range of immune cells and suppressing the antitumor activity of CD4^+^ T cells, CD8^+^ T cells, and NK cells (5, 95–99). In addition to these functions, tumor-resident Treg cells can promote tumor growth, angiogenesis, and metastasis. Treg cells also exert antitumor effects by inhibiting the immune response in the tumor microenvironment. In many cases, rich Treg cell infiltration into tumor microenvironment is correlated with poor prognosis (95). However, Treg cells play controversial roles in some cancers, in which abundance Foxp3^+^ Treg cells promised relatively good prognosis. A recent study found that in colorectal cancer, functionally distinct subsets of tumor-infiltrating Foxp3^+^ T cells contribute in opposing ways to measuring outcomes (100). Dissection of the pathways regulated by tumor-resident Treg cells is critical for immunotherapies that aim to modulate Treg cells in cancer.

## Conclusion

Recent studies have provided new insights into Treg cell regulation and homeostasis. Dynamic homeostatic processes maintain the diverse pool of Treg cells and preserve the number of Treg cells within steady-state conditions. Treg cells have two origins and can be divided into functional subsets. Tissue-resident Treg cells are a relatively new subtype; thus, it is not surprising that there are some questions that remain unanswered. Here, we highlight three questions that need to be explored. First, which type of Treg cells play a predominant role in the regulation of the immune response compared with non-resident Treg cells? Second, are tumor-specific Treg cells mainly derived from tissue-resident Treg cells or from tTreg cells and pTreg cells? Third, can Treg cells be classified into new subsets, and can the degree of infiltration of these subpopulations contribute to disease prognosis?

Regulatory T cell homeostasis is governed by a number of factors. In this review, we focused on the exosome- and ncRNA-mediated regulation of Treg cell homeostasis. The study of Treg cell-derived exosomes is a relatively new area of Treg cell biology. The exosome output by Treg cells changes with cell status and reflect intracellular events. Exosomes, therefore, provide an enriched pool of information and could be considered to be potential biomarkers. In addition to the effects of Treg cell-derived exosomes on immune responses, exosomes could be used as therapeutic agents in various conditions. ncRNAs are crucial to the homeostasis of Treg cells. There are many studies of miRNA and lncRNA, but the roles and mechanisms of other classes of ncRNAs, including circRNA, in the regulation of Treg cells remain unclear and will require further study.

## Author Contributions

All the authors listed have made a substantial, direct, and intellectual contribution to the work and approved it for publication.

## Conflict of Interest Statement

The authors declare that the research was conducted in the absence of any commercial or financial relationships that could be construed as a potential conflict of interest.
